# Assessing the Efficacy of a Novel Massive Open Online Soft Skills Course for South Asian Healthcare Professionals

**DOI:** 10.1007/s10916-024-02051-5

**Published:** 2024-03-21

**Authors:** Aditya Mahadevan, Ronald Rivera, Mahan Najhawan, Soheil Saadat, Matthew Strehlow, G. V. Ramana Rao, Julie Youm

**Affiliations:** 1 University of California Irvine School of Medicine, Irvine, CA USA; 2 Department of Emergency Medicine, University of California Irvine Medical Center, Orange, CA USA; 3 University of Queensland-Ochsner School of Medicine, New Orleans, LA USA; 4 Department of Emergency Medicine, Stanford University School of Medicine, Stanford, CA USA; 5 Department of Emergency Medicine Learning Centre and Research, Emergency Management and Research Institute, Hyderabad, Telangana India; 6 Department of Medical Education, University of California Irvine School of Medicine, Irvine, CA USA

**Keywords:** South Asia, Online education, Medical education, Internet, Soft skills, Massive open online course, MOOC

## Abstract

In healthcare professions, soft skills contribute to critical thinking, decision-making, and patient-centered care. While important to the delivery of high-quality medical care, soft skills are often underemphasized during healthcare training in low-and-middle-income countries. Despite South Asia’s large population, the efficacy and viability of a digital soft skills curriculum for South Asian healthcare practitioners has not been studied to date. We hypothesized that a web-based, multilingual, soft skills course could aid the understanding and application of soft skills to improve healthcare practitioner knowledge, confidence, attitudes, and intent-to-change clinical practice.In September 2019 a needs assessment observing soft skills practices was conducted in several Indian states. We developed a communication-focused soft skills curriculum that comprised seven 10-minute video lectures, recorded in spoken English and Hindi. Participants consisted of any practicing healthcare professionals and trainees in select South Asian countries age 18 and over. Participant knowledge, confidence, attitudes, and intent-to-change clinical practice were evaluated using pre- and post-course tests and surveys. Statistical analyses were performed using STATA and SPSS.From July 26, 2021 to September 26, 2021, 5750 registered and attempted the course, 2628 unique participants completed the pre-test, and 1566 unique participants completed the post-test. Participants demonstrated small but statistically significant gains in confidence (𝑝<0.001), attitudes toward course topics relevance (𝑝<0.001), and intent-to-change clinical practice (𝑝<0.001). There was no statistically significant gain in knowledge. A digital soft-skills massive open online course for healthcare practitioners in South Asia could serve as a viable approach to improve the quality of soft skills training in low-to-middle income countries.

## Introduction

Soft skills are loosely defined as non-technical cognitive and interpersonal skills, such as teamwork and interpersonal communication, which complement profession-specific skills such as scientific knowledge and technical expertise [1, 2]. In healthcare professions, soft skills enhance critical thinking, decision-making, and patient-centered care, which are paramount to quality healthcare delivery and favorable patient outcomes; yet, healthcare educators have traditionally placed a greater emphasis on profession-specific skills [3, 4]. Prior studies assessing soft skills training across multiple medical universities found limited emphasis on teamwork and professional communication [5, 6].

Focus on soft skills education is especially important since communication is implicated in many unintended medical errors [7]. A study focused on low-and-middle-income countries (LMICs) determined that approximately 1.9 million people die each year due to poor quality care secondary to ineffective patient-practitioner communication and underdeveloped soft skills [8]. Directed educational interventions can effectively address these deficiencies. Prior interventions to address these deficiencies have led to measurable improvements in patient satisfaction, employee satisfaction, and patient outcomes [9]. In South Asia, a study of a non-technical skills curriculum for paramedics yielded increases in confidence and body language as measured by blinded, third-party evaluators [10]. Additionally, a study evaluating a basic communication course for 48 Indian medical students found improvements in their post-course clinical performance during simulated patient encounters [11].

Implementing soft skills instruction in LMICs is challenging due to geographical distances, cultural differences, language barriers, and a paucity of regional subject matter experts [12, 13]. While traditional classroom-based teaching methods may be effective for delivering such content, this approach alone is time-intensive, and lacks scalability. Burgeoning access to the Internet has enabled online instruction to become a reliable, effective, inexpensive, and scalable platform for addressing this need [14, 15]. This was demonstrated during the COVID-19 pandemic, which prompted a shift to remote medical educational strategies [16, 17]. Internet access and availability have increased globally, particularly in LMICs, making internet access less of a barrier to digital education [18].

While virtual soft skills education has shown promise in high-income countries (HICs) including the United States, it has yet to be studied amongst South Asian healthcare practitioners [19, 20]. In recent years, massive open online courses (MOOCs) have emerged as a potential approach to provide remote education to large amounts of participants [15]. Our objective was to assess the impact of a novel massive open-online, multi-language, video-based soft skills course developed specifically for South Asian healthcare practitioners. We hypothesized that our web-based, multilingual, massive open-online soft skills course could improve South Asian healthcare practitioner knowledge, confidence, attitudes, and intent-to-change clinical practice, regarding their understanding and application of soft skills.

## Materials and Methods

### Ethics Statement

Following completion of the exempt self-determination tool, this study met criteria for and was granted Institutional Review Board exemption by the UC Irvine IRB.

### Study Design

This study was designed as a single group pre-and post-test design quasi-experimental study with no control group.

### Setting

The open-access online course was promoted by regional healthcare agencies (e.g., EMRI Green Health Services in India) through modalities such as email and printed newsletters in selected South Asian countries, including India, Pakistan, and Myanmar. Data was collected from July 26, 2021 to September 26, 2021.

### Participants

Participants consisted of any practicing healthcare professionals and trainees in select South Asian countries over the age of 18, including nurses, prehospital care providers, and physicians.

### Interventions

In September 2019, we conducted an in-depth needs assessment of soft skills practices in India by observing and interviewing various healthcare practitioners (in the Indian states of Haryana, Uttar Pradesh, Delhi, and Telangana) working in various healthcare settings such as outpatient clinics, hospitals, ambulances, and medical training institutions. We created an educational framework that addressed literature-recognized deficiencies in practitioner training and commonly observed challenges in patient care delivery. We identified six key elements of soft skills: language barriers, personal biases and stigma, nonverbal communication, paraverbal communication, active listening, and step-by-step clinical communication. We then filled out the course framework using relevant examples from a variety of healthcare settings. The intervention comprised an online pre-course survey and pre-test, seven digitally available 10-minute video lectures, recorded in spoken English and Hindi, and an online post-course survey and post-test.

Participants accessed the study site through an anonymous link, generated a unique identifier, and digitally consented. After completing the pre-course survey and pre-test, participants were emailed a direct link to the online training videos. After attesting that they had watched all seven training videos, participants were emailed a second link to the post-course survey and post-test in which they re-entered their unique identifier. The study was considered completed if participants finished the pre- and post-course survey and test. Using participants’ unique identifiers, the pre- and post-course surveys and tests were matched to form the “matched participants”. Completed non-duplicate pre-tests and completed non-duplicate post-tests unable to be matched by participants’ unique identifiers comprised the “unmatched participants”.

### Outcomes

Outcomes of the study were as follows: changes in participant knowledge, confidence, attitudes, and intent-to-change clinical practice, measured before and after the soft-skills education course.

### Data Sources/Measurements

QualtricsXM was used to create the study’s questionnaires and collect data. Pre- and post-course surveys used a 5-point Likert scale to assess prior soft skills exposure and/or training, confidence with the course topics, attitudes towards the course topics, and intent-to-change clinical practice.

The pre-course survey collected non-identifying demographic information about participants and assessed the participants’ previous soft skills training, attitudes, and confidence, using the five-point Likert scale. The pre-test included 13 multiple-choice test questions, drafted by the course creators and vetted for face and content validity.

Using the information gathered from the needs assessment, seven 10-minute training videos were recorded in both spoken English and Hindi and subtitled in Hindi using screen capture software (Camtasia®, TechSmith Corporation, USA). Hindi translations were back-translated and verified by a professional interpreter.

The post-course survey re-assessed participant attitudes, confidence, and intent-to change clinical practice; additionally, participants were asked to rate the relevance of the course content, the usefulness of Hindi translation, and if they would recommend this course to colleagues. The post-test comprised 26 randomized multiple-choice questions. Half of these questions were the same as the pre-test, and the other half were designed to apply the concepts learned in the videos to novel clinical situations and questions.

### Study Size and Design

An appropriate sample size (200) was determined using a Cohen’s d of 0.2 for a within-subjects design with an alpha of 0.05 and power of 0.8. As a within-subjects design, participants were not assigned. There was no blinding for participants. The unit of analysis was the change in each individual participant’s knowledge from before intervention to after intervention.

### Statistical Methods

Frequencies are presented as N (%), and continuous variables as mean or median and 95% confidence interval or Inter Quartile Range (IQR) as relevant. We used the chi-square test to compare the distribution of categorical variables across groups. Knowledge gain was analyzed by two approaches. For participants whose pre- and post-test surveys were able to be matched (*N* = 1062), we compared the change in their knowledge score from pre-intervention to post-intervention by using paired samples t-test, calculated based on 13 identical questions that appeared in both the pre- and post-test. The effect size of the educational intervention was reported using Cohen’s d.

For participants whose pre- and post-test surveys were able to be matched (*N* = 1062), expressed intention to change, attitude, and expressed gain in confidence were compared after intervention compared to pre-intervention by using Wilcoxon signed rank test. Additionally for all matched participants (*N* = 1066), post-course satisfaction was determined based on a Likert scale survey.

## Results

**Fig. 1:**
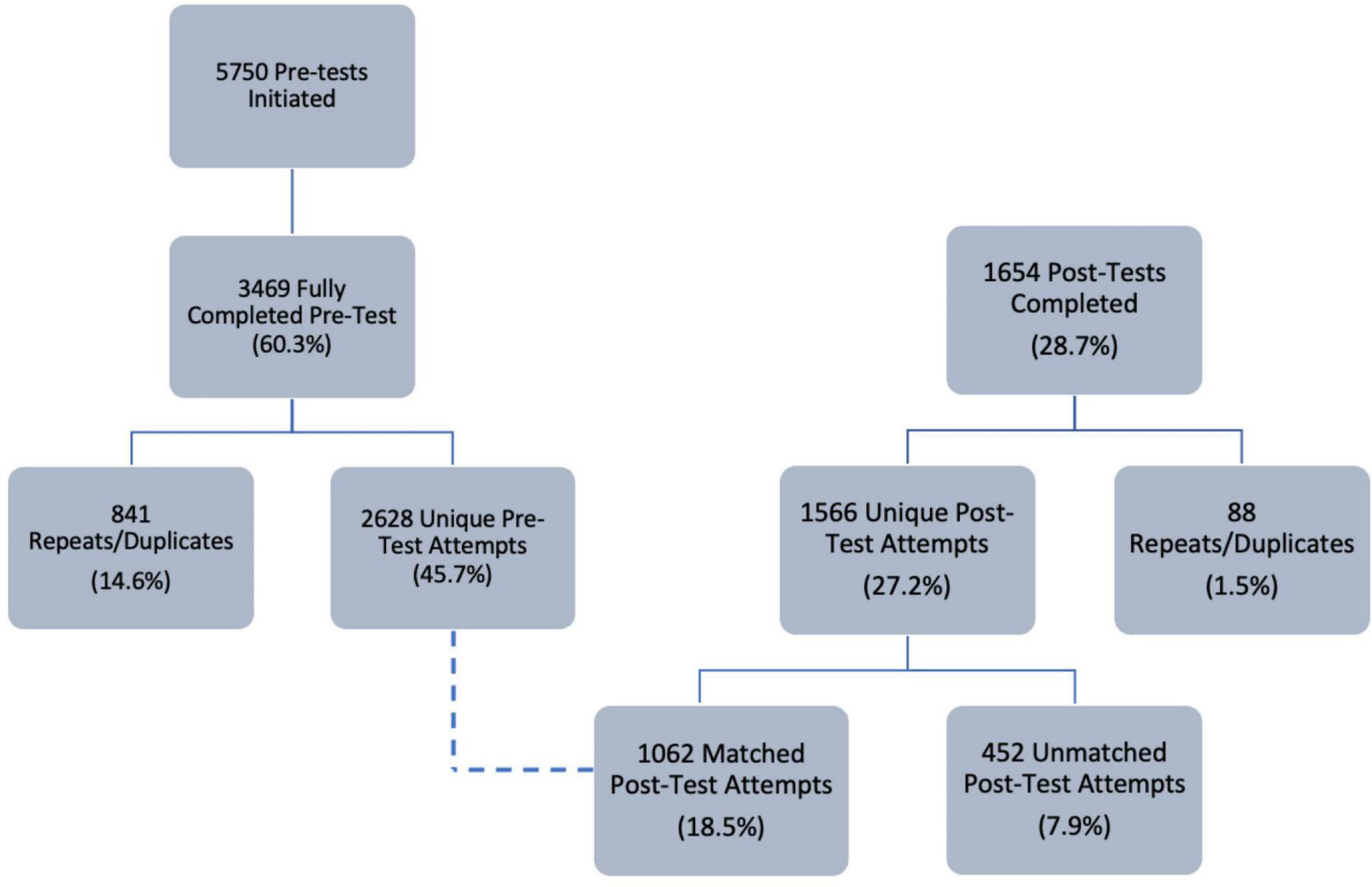
Course completion

Between July 26, 2021, and September 26, 2021, 5417 participants completed the consent form and created a unique identifier and 2628 participants fully completed the pre-test. 1566 unique participants (27.2%) fully completed the post-test, which we refer to as the unmatched participants (Fig. 1). We had 1062 unique matched participants who fully completed the pre and post-tests, which we refer to as the matched participants (Fig. 1).

The age range of those who completed the course was 20–53 years, with a median age of 31 (IQR 27.0–38.0). Females accounted for 13.6% (*N* = 213) of those completing the course. The time to complete the course ranged from 4.5 min to 13 days (IQR 26.3- 118.0 min) with a median of 50.6 min.

## Participants by Practitioner type

Prehospital care providers (paramedics and EMTs) accounted for 42.0% of participants (*N* = 657), the highest percentage of those completing the course, whereas physicians accounted for 1.9% (*N* = 29) (Table 1). 92% of those completing the course worked directly with patients (*N* = 1441).

**Table 1:** Participant demographics

	Variable	Matched Participants (Matched Post-Test Attempts) (*n* = 1062)	%	Unmatched Participants (Unique Post-test Attempts) (*n* = 1566)	%
Country	India	1030	97.0%	1514	96.7%
	Nepal	0	0.0%	4	0.3%
	Bhutan	0	0.0%	1	0.1%
	Myanmar	4	0.4%	4	0.3%
	Pakistan	3	0.3%	6	0.4%
	Other	25	2.4%	37	2.4%
Gender	Male	933	87.9%	1352	86.3%
	Female	129	12.1%	213	13.6%
	Other	0	0.0%	1	0.10%
Primary Spoken Language	Hindi	118	11.1%	208	13.3%
	English	10	0.9%	26	1.7%
	Bengali	1	0.1%	1	0.1%
	Marathi	2	0.2%	7	0.4%
	Telugu	2	0.2%	9	0.6%
	Tamil	2	0.2%	4	0.3%
	Gujarati	905	85.2%	1265	80.8%
	Kannada	2	0.2%	3	0.2%
	Odia	0	0.0%	0	0.0%
	Malayalam	0	0.0%	0	0.0%
	Punjabi	1	0.1%	1	0.1%
	Nepali	0	0.0%	3	0.2%
	Burmese	4	0.4%	4	0.3%
	Dzongkha	0	0.0%	1	0.1%
	Urdu	3	0.3%	9	0.6%
	Other	12	1.1%	25	1.6%
Profession	Community health worker	0	0.0%	0	0.0%
	Health-related researcher	0	0.0%	0	0.0%
	Nurse	0	0.0%	0	0.0%
	Nurse midwife	0	0.0%	0	0.0%
	Nursing assistant	0	0.0%	0	0.0%
	Paramedic or EMT	0	0.0%	0	0.0%
	Pharmacist	0	0.0%	0	0.0%
	Community health worker	79	7.4%	212	13.5%
	Health-based researcher	22	2.1%	33	2.1%
	Nurse	28	2.6%	53	3.4%
	Nurse Midwife	0	0.0%	5	0.3%
	Nursing Assistant	21	2.0%	52	3.3%
	Paramedic or EMT	561	52.8%	657	42.0%
	Pharmacist	1	0.1%	6	0.4%
	Physician	12	1.1%	29	1.9%
	Physician assistant or nurse practitioner	4	0.4%	9	0.6%
	Public health practitioner	30	2.8%	81	5.2%
	Student	12	1.1%	17	1.1%
	Traditional or complementary medicine practitioner	3	0.3%	3	0.2%
	Other	289	27.2%	409	26.1%
Patient Communication	Yes	966	91.0%	1441	92.0%

## Geographic Distribution of Participants

Of those completing the course, the vast majority (96.7%, *N* = 1514) were from India and the most common native language spoken was Gujarati (80.8%, *N* = 1265) (Table 1).

## Knowledge Gain

Matched participants did not demonstrate a statistically significant knowledge gain (d = 0.047, 𝑝= 0.131) when comparing 13 pre-test questions with the matching 13 post-test questions (Table 2).

**Table 2:** Change in knowledge amongst participants

**Cohort**	**Pre-Test Attempts**	**Post-Test attempts**	**Mean Difference**	**Standard Error**	𝑝 **value**	**Cohen’s d**
Matched	1062	1062	-0.09	0.06	0.131	0.047

## Attitudes

Participants rated the relative importance of each topic before and after course completion. Participants demonstrated positive changes regarding their attitudes in all six assessed subject areas: language barriers, stigmas, nonverbal communication, paraverbal communication, active listening, and step-by-step clinical communication (𝑝<0.001) (Table 3).

**Table 3:** Attitude change from pre intervention to post intervention

Variable	N	Mean	Median	IQR	SD	Negative rank	Positive rank	Wilcoxon statistics	𝑝 -value
Language Barriers PRE	1062	3.9	4.0	1.0	1.07	143	239	-3.75	< 0.001
Language Barriers POS	1062	4	4.0	1.0	1.1				
Stigma PRE	1062	3.7	4.0	2.0	1.2	126	259	-6.16	< 0.001
Stigma POS	1062	3.9	4.0	1.0	1.13				
Nonverbal Communication PRE	1062	4.1	4.0	1.0	0.99	99	191	-5.75	< 0.001
Nonverbal Communication POS	1062	4.3	4.0	1.0	0.87				
Paraverbal Communication PRE	1062	4.2	4.0	1.0	0.95	104	185	-4.09	< 0.001
Paraverbal Communication POS	1062	4.3	4.0	1.0	0.9				
Active Listening PRE	1062	4.2	4.0	1.0	0.92	99	179	-4.59	< 0.001
Active Listening POS	1062	4.3	4.0	1.0	0.87				
Clinical Communication PRE	1062	4.2	4.0	1.0	0.92	99	178	-4.79	< 0.001
Clinical Communication POS	1062	4.3	4.0	1.0	0.84				

## Confidence

Participants improved their confidence in all six assessed subject areas: language barriers, stigmas, nonverbal communication, paraverbal communication, active listening, and step-by-step clinical communication (𝑝<0.05) (Table 4).

**Table 4:** Confidence change from pre-intervention to post-intervention

Item Variable	N	Mean	Median	IQR	SD	Negative rank	Positive rank	Wilcoxon statistics	𝑝-value
Language Barriers PRE	1062	4.0	4.0	1.0	0.97	135	209	-2.94	0.003
Language Barriers POS	1062	4.1	4.0	1.0	0.98				
Stigma PRE	1062	4.1	4.0	1.0	0.99	119	223	-4.98	< 0.001
Stigma POS	1062	4.2	4.0	1.0	0.95				
Nonverbal Communication PRE	1062	4.1	4.0	1.0	1.02	105	209	-5.32	< 0.001
Nonverbal Communication POS	1062	4.2	4.0	1.0	0.95				
Paraverbal Communication PRE	1062	4.2	4.0	1.0	0.89	118	178	-2.68	0.007
Paraverbal Communication POS	1062	4.3	4.0	1.0	0.91				
Active Listening PRE	1062	4.2	4.0	1.0	0.95	120	197	-4.25	< 0.001
Active Listening POS	1062	4.3	4.0	1.0	0.9				
Clinical Communication PRE	1062	4.1	4.0	1.0	0.95	104	206	-4.77	< 0.001
Clinical Communication POS	1062	4.2	4.0	1.0	0.92				

## Training and Intent to Change

Participants answered questions regarding their improvement in prior knowledge and intent to apply these learned skills to future clinical practice. Participants reported high levels of intent to change clinical practice across all six measured subject areas: language barriers, the role of stigmas, nonverbal communication, paraverbal communication, active listening, and step-by-step clinical communication (𝑝<0.001) (Table 5).

**Table 5:** Intent to change from pre intervention to post intervention

ItVariable	N	Mean	Median	IQR	SD	Negative rank	Positive rank	Wilcoxon statistics	𝑝-value
Language Barriers PRE	1062	4	4.0	1.0	1.14	130	266	-7.85	< 0.001
Language Barriers POS	1062	4.3	4.0	1.0	0.9				
Stigma PRE	1062	4	4.0	1.0	1.12	103	270	-9.6	< 0.001
Stigma POS	1062	4.3	4.0	1.0	0.83				
Nonverbal Communication PRE	1062	4	4.0	1.0	1.13	112	277	-9.01	< 0.001
Nonverbal Communication POS	1062	4.3	4.0	1.0	0.89				
Paraverbal Communication PRE	1062	4.1	4.0	1.0	1.05	111	233	-7.62	< 0.001
Paraverbal Communication POS	1062	4.3	4.0	1.0	0.82				
Active Listening PRE	1062	4.1	4.0	1.0	1.05	108	229	-7.44	< 0.001
Active Listening POS	1062	4.3	4.0	1.0	0.82				
Clinical Communication PRE	1062	4.1	4.0	1.0	1.04	101	219	-7.34	< 0.001
Clinical Communication POS	1062	4.3	4.0	1.0	0.82				

## Satisfaction

**Fig. 2:**
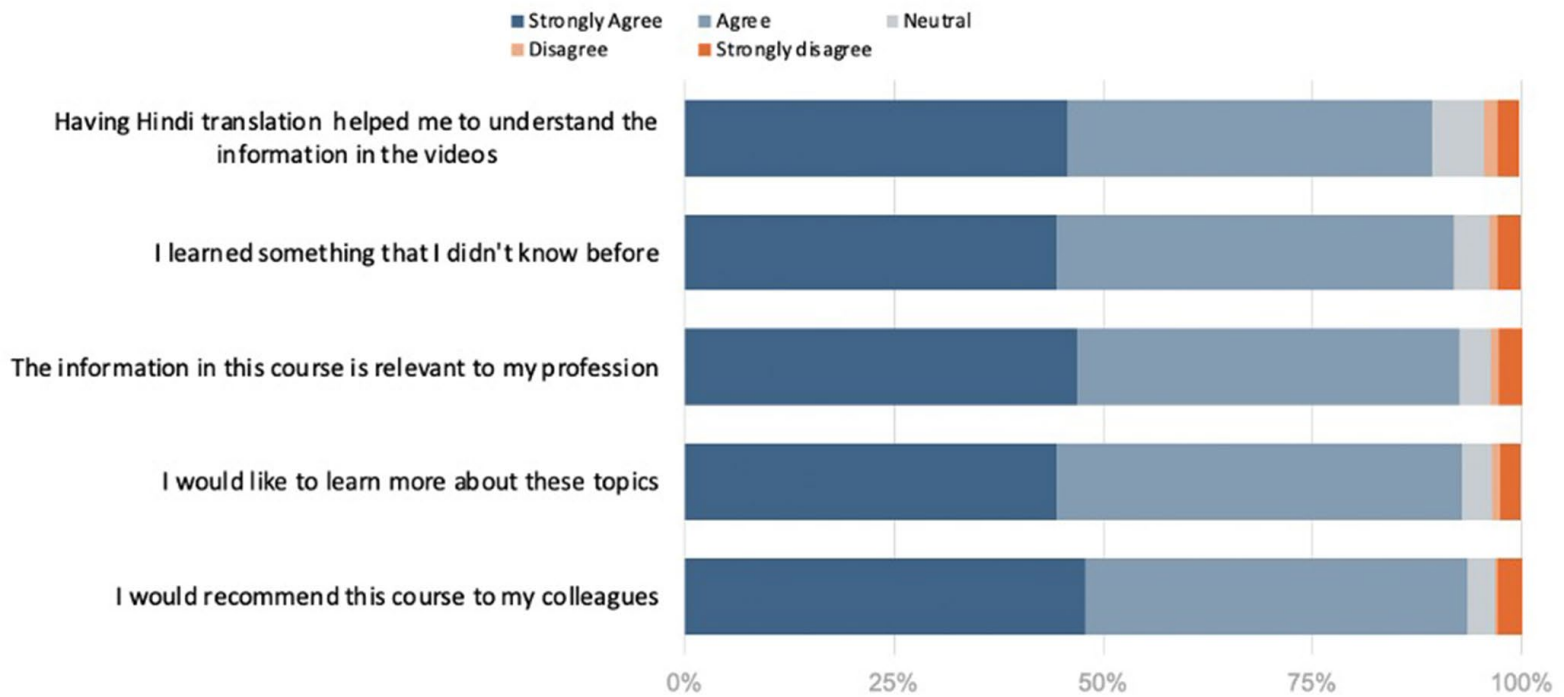
Post-course satisfaction

Regarding the utility of Hindi translation, 89.3% of participants (95%CI [87.3%,90.4%], *N* = 1062) agreed or strongly agreed that access to translated text and subtitles improved their understanding of course content (Fig. 2). In addition, 91.8% of participants (95%CI [90.4%,93.1%], *N* = 1062) agreed or strongly agreed that they learned something new, 92.5% (95%CI [90.0%,92.7%], *N* = 1062) found the course relevant, 92.8% expressed a desire to learn more (95%CI [90.0%,92.8%], *N* = 1062), and 93.5% would recommend the course to colleagues (95%CI [91.1%,93.7%], *N* = 1062).

## Discussion

This study evaluated the efficacy of a web-based, multilingual, massive open-online soft skills course, developed specifically for practicing South Asian healthcare practitioners. To our knowledge, this was the first study to evaluate the efficacy of a massive open online, multi-language, video-based soft skills course, developed specifically for practicing South Asian healthcare practitioners during the COVID-19 pandemic. Practitioners from a diverse array of clinical backgrounds enrolled in the course with a greater-than-expected completion percentage [21]. Regarding taught soft skills, course participants demonstrated detectable attitude changes, increased confidence, and substantial intent to modify their clinical practice. Of equal importance, participants valued the overall course and would recommend it to their colleagues.

While the course had representation from 5 countries, 12 healthcare professions, and speakers of over 15 languages, our cohort was skewed as primarily male, Hindi-speaking, and prehospital care providers. This gender breakdown is likely related to the over-representation of men in the Indian healthcare workforce [22]. The predominance of prehospital care providers may have reflected strong representation from EMRI Green Health Services (GHS), India’s largest ambulance service. Our course completion rate of 27.2% was more than double the average completion rate of other massive-open online courses (MOOCs), attesting to interest in and desire for soft-skills training in India [21].

Knowledge gain amongst our matched participants was not significantly increased. This may be attributed to insufficient time spent reviewing the course content. While our interface required participants to click through each individual video in chronological order to reach the post-survey, we could not verify that participants viewed all 71 min of course content; in fact, our median time to course completion was 50.6 min, 20 min less than the total duration of all course videos. Similarly, excessive lecture time in other MOOCs has been linked to course dropout and poor engagement [21]. Additional strategies could be employed to increase participant engagement, including “chunking” course content into smaller videos, adhering more strongly to Mayer’s Multimedia Principles, and exploring video tools that enable integration of interactive elements [23].

Furthermore, as a course broadly targeted toward all healthcare workers, the intervention inherently may not have appealed directly to all practitioner types. This was especially true of the clinical vignettes in the pre and post-test questions as certain provider types were likely more familiar with the clinical scenarios and skill areas tested.

Participants showed significant changes in attitudes, gains in confidence, and intent to change clinical practice in all six subject areas: language barriers, stigmas, nonverbal communication, paraverbal communication, active listening, and step-by-step clinical communication. These survey findings were consistent with prior literature in healthcare education [24]. While some participants received prior training in these soft skill areas, their survey results consistently affirmed their desire to further develop these soft skills competencies, consistent with prior literature [25, 26]. Finally, participants recommended the course as clinically relevant to their peers, consistent with prior digital interventions [26]. While the large sample size of this study may have contributed to the statistical significance of small differences in survey results, these findings add to a growing body of literature suggesting that beyond knowledge gain, soft skill interventions targeting healthcare workers can significantly alter their attitudes and perceptions, potentially yielding changes in their clinical practice. While our study was conducted online on a significantly larger scale than the aforementioned studies, the virtual interface did not dampen participant interest in, or enthusiasm toward learning about soft skills.

## Limitations

While valuable as the first of its kind in South Asia, this study had some important limitations. As mentioned previously, we could not ensure that participants watched the video modules in their entirety. Additionally, the proportion of prehospital care providers in our sample makes it challenging to extrapolate our findings to all classes of healthcare practitioners in South Asia. As the course materials were translated only into Hindi, this may have limited uptake in non-Hindi-speaking South Asian countries. Finally, while knowledge gain was our primary outcome measure, it may not directly affect the quality of clinical care.

## Generalizability

The significant rate of preventable global mortality attributed to underdeveloped patient-practitioner communication highlights the need for further soft skills training in LMICs, where these skills may not have been taught or emphasized [5, 6, 8]. Our digital curriculum was the first of its kind, contextualized for South Asian healthcare practitioners at all training levels, and created in conjunction with South Asian healthcare practitioners after a lengthy field-based needs assessment. Effective educational curricula should ideally incorporate language-accessibility and cultural context. We addressed this by providing simultaneous English-Hindi narration/subtitling and culturally-relevant clinical examples drawn from real-life clinical settings [25].

While numerous barriers to in-person education exist in LMICs, utilization of a virtual platform may allow for an effective yet scalable approach to address these challenges [12, 13]. Given that our participants reported intent to change their clinical practice, this novel investigation supports the potential viability of virtual soft skills didactic interventions for healthcare practitioners in LMICs [14, 15].

Future studies should continue to explore the impact of virtual soft skills curricula abroad on healthcare outcomes beyond rote knowledge gain and attitude changes. Such studies should also explore the impact of time engaged with course content on knowledge gained. Adding an evaluation component through simulated clinical scenarios could assess a participant’s ability to apply their new knowledge. Substantial improvement in measured competencies would likely represent a significant step toward improving quality of care and reducing preventable morbidity and mortality in LMICs.

## Conclusion

Improvements in access to technology and increasing quality of free open-access medical education have demonstrated that MOOCS are a cost-effective, scalable way to train healthcare professionals. Furthermore, they can overcome substantial barriers like cost, geography, and language, that have previously hindered the development of healthcare systems in LMICs. However, to this point, virtual soft skills education has been underexplored and underutilized in South Asia, despite a clear need. Following the completion of our MOOC, study participants displayed a significant improvement in confidence, attitude, and intent-to-change clinical practice. In the future, MOOCs may be utilized to break down barriers and improve the quality of training in LMICs, not just for technical skills but soft skills as well.

## Data Availability

The data is stored in a secure, maintained enterprise cloud platform and is available on request.
